# Environmental Carcinogenesis and Transgenerational Transmission of Carcinogenic Risk: From Genetics to Epigenetics

**DOI:** 10.3390/ijerph15081791

**Published:** 2018-08-20

**Authors:** Ernesto Burgio, Prisco Piscitelli, Annamaria Colao

**Affiliations:** 1 European Cancer and Environment Research Institute (ECERI), 1000 Bruxelles, Belgium; erburg@libero.it; 2 Euro Mediterranean Scientific Biomedical Institute (ISBEM), 72023 Mesagne, Brindisi, Italy; 3 Department of Clinical Medicine and Surgery, University Federico II School of Medicine, 80138 Naples, Italy; colao@unina.it

**Keywords:** environmental carcinogens, cancer pathogenesis and mechanisms, epigenetics

## Abstract

The dominant pathogenic model, somatic mutation theory (SMT), considers carcinogenesis as a ‘genetic accident’ due to the accumulation of ‘stochastic’ DNA mutations. This model was proposed and accepted by the scientific community when cancer mainly affected the elderly, but it does not explain the epidemiological observation of the continuous increase in cancer incidence among children and young adults. Somatic mutation theory has been proposed for a revision based on the emerging experimental evidence, as it does not fully address some issues that have proven to be crucial for carcinogenesis, namely: the inflammatory context of cancer; the key role played by the stroma, microenvironment, endothelial cells, activated macrophages, and surrounding tissues; and the distorted developmental course followed by the neoplastic tissue. Furthermore, SMT is often not able to consider either the existence of specific mutations resulting in a well-defined cancer type, or a clear relationship between mutations and tumor progression. Moreover, it does not explain the mechanism of action of the non-mutagenic and environmental carcinogens. In the last decade, cancer research has highlighted the prominent role of an altered regulation of gene expression, suggesting that cancer should be considered as a result of a polyclonal epigenetic disruption of stem/progenitor cells, mediated by tumour-inducing genes. The maternal and fetal exposure to a wide range of chemicals and environmental contaminants is raising the attention of the scientific community. Indeed, the most powerful procarcinogenic mechanisms of endocrine disruptors and other pollutants is linked to their potential to interfere epigenetically with the embryo-fetal programming of tissues and organs, altering the regulation of the genes involved in the cell cycle, cell proliferation, apoptosis, and other key signaling pathways. The embryo-fetal exposure to environmental, stressful, and proinflammatory triggers (first hit), seems to act as a ‘disease primer’, making fetal cells and tissues more susceptible to the subsequent environmental exposures (second hit), triggering the carcinogenic pathways. Furthermore, even at the molecular level, in carcinogenesis, ‘epigenetics precedes genetics’ as global DNA hypomethylation, and the hypermethylation of tumor suppressor genes are common both in cancerous and in precancerous cells, and generally precede mutations. These epigenetic models may better explain the increase of cancer and chronic/degenerative diseases in the last decades and could be useful to adopt appropriate primary prevention measures, essentially based on the reduction of maternal-fetal and child exposure to several procarcinogenic agents and factors dispersed in the environment and in the food-chains, as recently suggested by the World Health Organization.

## 1. Introduction

It is well known that prenatal life is not fully protected in the uterus. However, it is only over the last decade that research has been focusing on the mechanisms and modalities of maternal and fetal exposure to a wide range of chemicals (i.e., endocrine disruptors), physical factors (such as ionizing radiations), and biological agents (i.e., viruses) that may induce potentially adaptive and predictive epigenetic modifications in the embryo-fetal genome, thus interfering with the programming of tissues and organs in an often irreversible way. Embryo-fetal cells are, by definition, epigenetically plastic. Indeed, it is during embryo–fetal development that trillions of cells that will constitute our body differentiate, acquiring their own specific epigenetic program based on the information reaching the fetus. Several studies have already documented that hundreds of epi-genotoxic molecules, present in the placentas and umbilical cords, interfere with cell and tissue programming. This field of investigation supports the Developmental Origins of Health and Disease theory (DOHaD), based on the concept that the ‘early life environment’ has remarkable consequences for the adult health [1]. In particular, it is suggested that fetal cells might undergo reactive epigenetic changes, which represent potentially adaptive-defensive responses to environmental factors (fetal programming). This could also account for the current, unremitting increase of endocrine–metabolic, cardiovascular, immunological, neurodevelopmental, and neurodegenerative diseases (epidemiological transition) [2]. Paradoxically, cancer has not been studied so far in this perspective, because of a dominant pathogenic model, the so-called somatic mutation theory (SMT), which considers cancer a sort of ‘genetic accident’ due to the accumulation of stochastic mutations. This model was proposed and accepted by the scientific community when cancer generally affected old people, but it does not fit anymore with the epidemiological observation of cancer’s significant increase in small children and young adults [3]. Usually, when a theoretical model based on simple assumptions (such as the theory of the accumulation of stochastic mutations during life) is no longer able to explain the observed phenomena, it is time to move towards new theories, by introducing new variables based on the emerging evidence. In this perspective, as we will see, cancer could be much better explained as an ‘epigenetic disease’. Indeed, infant cancer has registered one of the highest increase in incidence among all age groups and could represent the most emblematic consequence of a distorted fetal programming, showing peculiar characteristics also at the molecular level.

### 1.1. Reconsidering the Dominant Neo-Darwinian Model of Cancer

For almost half a century, the so-called somatic mutation theory has been the dominant paradigm to explain carcinogenesis and, in particular, the development of tumors related to environmental exposures (environmental carcinogenesis). According to this theory, cancer is considered a genetic accident, resulting from the progressive accumulation of random (stochastic) mutations of DNA [4].

Some authors have already pointed out that this molecular model is the transposition of the Neo-Darwinian paradigm [5] (which has dominated the field of evolutionary biology for over a century) within the biomedical field. The Neo-Darwinian paradigm is precisely based on the idea that the appearance of new species (macro-evolution) is the product of stochastic DNA mutations (microevolution) producing advantages (fitness) and being ‘rewarded’ by natural selection. In the field of carcinogenesis, instead of considering organisms and populations, scientists consider neoplastic cells as wrongly evolving entities inside a multicellular organism [6]. At this point, it is important to underline that, within the Neo-Darwinian model, mutations are supposed to emerge—usually in accidental way—in the single genome, while natural selection operates on phenotypes (i.e., populations). The formation of a new species does not actually require the development of new genes, but it only requires the accumulation of mutations within existing genes. Yet, it is natural selection, operating on the medium-long term, which guides the evolutionary process.

It is not the purpose of this work to address the main criticisms to the Neo-Darwinian model. It is enough to point out that some authoritative philosophers of science have already denied the scientific validity of such a theory, largely based on the pure concept of ‘natural selection’. Some evolutionary biologists and geneticists have focused their criticisms of Neo-Darwinism on what they saw as “unrealistic, ‘atomistic’ models of both gene selection and trait evolution” [7]. Other scientists put at the center of the scene the basic molecular variations (genetic, epigenetic, and genomic) and the modalities of their formation, as in the absence of variations, natural selection would have nothing to act upon [8].

With regard to the most recent evolutionary theories, we can discuss the so-called Evo-Devo theory (evolutionary–developmental biology), which analyzes the structure of the genome, in particular the embryonic and fetal ones (epigenetically extremely plastic), from an evolutionary perspective. It hypothesizes that some epigenetic modifications of the genes controlling the early embryonic development (in particular the so-called master genes, remarkably conserved in evolution from insects to mammals) would give rise to new characteristics in the adult phenotype, eventually being transmitted to the descendants [9]. It is important to stress the revolutionary significance of this theory that, according to many biologists, transcends the narrow horizon of the Neo-Darwinian theory. Indeed, the Evo–Devo theory reconnects, after decades of separation, embryology (developmental biology/ontogeny) and evolutionary biology (phylogeny). Moreover, it reverses the traditional ‘theory of recapitulation’—generally defined by Ernst Haeckel’s sentence “ontogeny recapitulates phylogeny”—proposing ontogeny (namely embryonic development) as a sort of laboratory of evolution, where nature tests new and different living forms (morphogenesis). It is important to note that, according to this, the appearance of new characters within a population and even the advent of new species would not require changes in the sequence of genes (mutations) but could derive from simple changes in their regulation (epimutations) [10]. It should be clear that, in this perspective, the focus of the evolutionary process shifts towards genomic ‘variations’ and the direct production of new characteristics. This overshadows natural selection, which is a sort of *deus ex machina* in the Darwinian paradigm, while for its opponents it is the weak point of the model.

### 1.2. Weak Points of the Somatic Mutation Theory and Contribution of Epigenetics in Better Understanding Carcinogenesis

With specific regard to the issue of carcinogenesis, the SMT model has been criticized for decades [11], and requires a revision based on new experimental studies [12]. Indeed, the SMT fails to recognize the role of inflammation in carcinogenesis [13], and the key role played not only by the stroma [14], the microenvironment [15], endothelial cells [16], activated macrophages [17], and surrounding tissues [18], but also the distorted developmental course followed by the neoplastic tissue [19]. Furthermore, SMT is often not able to prove either the existence of specific mutations resulting in a well-defined neoplastic type [20], nor a clear relationship between mutations and tumor progression [21]. Moreover, the SMT does not clarify the action of non-mutagenic carcinogens [22], the unpredictability of tumor phenotypes, and the carcinogenic process itself [23]. Lastly, it is noteworthy that some benign tumors, such as lipomas and adenomas, are characterized by a significant number of mutations coinciding with those typical of the homologous neoplastic forms, liposarcomas and adenocarcinomas [24].

Instead, in the last decade, cancer research has highlighted the prominent role of an altered epigenetic regulation of gene expression [25]. Feinberg et al. had already suggested, in 2006, that epigenetics and genetics should be combined to achieve a better understanding of cancer as a result of “a polyclonal epigenetic disruption of stem/progenitor cells, mediated by tumor-progenitor gene” [21]. In general, we can say that epigenetics precedes genetics in carcinogenesis. Actually, in cancerous and precancerous cells, global DNA hypomethylation (particularly of regulatory sequences) leads to genomic instability, loss of imprinting (LOI) [26], activation and mobilization of retrotransposons [27], transcription of proto-oncogenes [28] and genes encoding proteins involved in genomic instability [29], and metastasis [30]. Still, the hypermethylation of the promoter sequences of various tumor suppressor genes (TSGs) causes their transcriptional silencing [31]. Moreover, recent cancer genome analyses have identified an impressive number of epigenetic enzymes that are deregulated in many types of cancer [32], whereas most miRNAs have different profiles in cancer compared with normal tissues and may act as oncogenes and tumor suppressors [33].

Bert Vogelstein conceived some of the most well-known and universally accepted formulations of the SMT model, according to which, the progressive accumulation of random ‘driver mutations’ of specific genes that control the cell cycle, proliferation, and programmed cell death (proto-oncogenes, tumor suppressor genes, and caretaker and gatekeeper genes), causes a deregulation of some key molecular pathways and the neoplastic transformation [34]. It is on this basis that cancer has still been recently framed as a genetic accident [35]. One of the most, if not the most cited articles concerning the SMT model is the that by Hannah and Weinberg, concerning the so-called ‘hallmarks of cancer’. Starting from the basic concept of the stochastic driver mutations in oncogenes and/or tumor suppressor genes, these authors enumerate a series of functional modifications typical of the neoplastic clone, namely: the insensitivity to growth-inhibitory (antigrowth) signals, the tendency to proliferate even in the absence of specific growth signals, the ability to evade apoptosis and to produce its own vascular system (angiogenesis), and the tendency to colonize the whole organism (metastasis) [36]. About ten years later, the same authors have expanded the list of hallmarks of cancer [37], and other scientists have further enriched their basic scheme by proposing a series of ‘metabolic hallmarks’, which are specific to the cell and the neoplastic clone [38].

One of the most intriguing criticisms of this model was proposed by a molecular biologist, Yuri Lazebnik, who pointed out that almost all of the hallmarks proposed by Hannah and Weinberg are not specific for cancer, being present in most of the benign tumors and in systemic inflammatory diseases, such as psoriasis and mononucleosis [39]. The only true hallmark of cancer is the most dangerous one, which usually determines the death of the organism itself, the tendency to metastasize. This criticism is highly relevant, as the metastatic process, rather than being the product of specific DNA mutations, is the consequence of the reactivation of an embryo-fetal program that allows cells to change their epigenetic layout. This allows neoplastic cells to convert themselves from stable epithelial cells into mesenchymal cells, capable of migrating to other body sites (epitelial mesenchimal transition—EMT). Moreover, both the embryonic and the neoplastic cells, once they have reached the destination site, tend to acquire a further molecular pathway (specular to the previous one), which allows them to regain their original epigenetic program and to anchor to the new tissue (mesenchimal epithelial transition—MET) [40]. The meaning of this should be evident; the most important and typical molecular and functional modifications induced by the neoplastic process are not the result of stochastic DNA mutations, but of the regain of specific epigenetic modifications, typical of the embryo-fetal period. In this context, it is also interesting to note that other fundamental characteristics of the neoplastic cell, such as the activation of the mobile sequences [41], the oncogenes [42], and the telomerases that contribute to the immortalization of the neoplastic cells [43] are not the result of DNA mutations, but of epigenetic modifications typical of the embryo–fetal period. Furthermore, the activation of Long Interspersed Element-1 (LINE-1) retrotransposon increases the risk of epithelial–mesenchymal transition and metastasis [44]. Despite all of this evidence, the majority of scientists persist in their efforts to find the driver mutations that trigger both cancer and metastasis. A complex evolutionary molecular approach (rigorously Darwinian) has been developed to study, without too much success so far, the sequential progression of mutations [45]. Surprisingly, the mutations potentially associated with metastasis do not appear late, as was expected in the context of the SMT theory [46]. On the contrary, it seems increasingly clear that the appearance of the ‘driver mutations’ is not only typical of the initial phases, but also occurs in the subsequent phases of the neoplastic process [47].

The literature discussed so far questions the basic concepts of the current carcinogenic paradigm, based on SMT and cancer hallmarks. In the following section, we will argue that the adoption of an Evo–Devo model could allow us to move in a decisive step towards understanding carcinogenesis.

### 1.3. Towards an Evo–Devo Model in Carcinogenesis

In the middle of the 19th century, some German pathologists of Virchow’s entourage had already noticed that neoplastic cells appeared, under a light microscope, very similar to embryonic cells. The pathologist had already assumed cancer to be an ontogenetic process gone away, starting from ectopic embryonic cells, or from embryonic residues, which—not having correctly completed the entire migratory path up to the definitive sites—received molecular signals from the surrounding tissues different from those necessary for their terminal differentiation, thus differentiating in abnormal way [48].

The model proposed by the 19th century pathologists found conclusive proofs from the studies concerning teratocarcinomas, which are neoplasms that develop from germ cells trapped in their migratory path, usually in the abdomen, therefore receiving ectopic molecular signals and undergoing uncontrolled proliferation. The result is a kind of dysontogenic draft of the whole organism, as the product of a distorted developmental path. Owing to this, teratocarcinomas are composed of tissues deriving from all the three of the embryonic layers of cells, the ectoderm, the endoderm, and the mesoderm (thyroid tissue, dental sketches, bone tissue, cartilage, muscle, etc.) [49].

At this point, we can ask the following: Is it possible to adopt such a ‘dysontogenic model’ in the field of carcinogenesis? Is it also possible to hypothesize and demonstrate that other neoplasms, particularly those characteristic of early childhood (which are dramatically increasing in recent decades), instead of being the result of a slow accumulation process of stochastic DNA mutations, recognize an epigenetic (and dysontogenic) origin in the embryo–fetal period? This has already been proven, not only for cancer types deriving from embryonic drafts—such as neuroblastoma, rabdiomiosarcoma, retinoblastoma, and nefroblastoma (better known as Wilms tumor)—but also for some tumors typical of adulthood, such as vaginal adenocarcinoma, breast carcinoma, and leiomyosarcoma.

We did not choose these three neoplasms by chance, and it is certainly not accidental that the cancer types that have already been demonstrated to have dysontogenic origins are the neoplasms of the endocrine system. Actually, scientists hypothesized a dysontogenic/embryo–fetal origin for these cancers in the context of the research concerning maternal and fetal exposure to epigenotoxic molecules. The latter represent procarcinogenic rather than mutagenic substances, belonging to the increasingly wider category of endocrine disruptors, which are mimetic molecules able to interfere in the adult on the endocrine system and to act—in the embryo or in the fetus—as morphogens or signal molecules that interfere with the differentiation processes and the right development of the organism in the very first steps of life. The first emblematic case of a possible dysontogenic and therefore carcinogenic action certainly was the DES-tragedy (diethylstilbestrol). Diethylstilbestrol (DES) was a synthetic nonsteroidal estrogen, advertised as “completely safe” for everyone, including mother and child, “even during pregnancy”, and was administered from 1940 to 1971 to hundreds of thousands of pregnant women. Unfortunately, DES was revealed to be a powerful transgenerational endocrine disrupter, causing the onset of urogenital malformations and of very rare vaginal adenocarcinomas in the daughters of the exposed women. Moreover, DES exposure determined a wide variety of developmental disturbances in the offspring, including urogenital abnormalities, birth defects, infertility, altered gender behavior, an increased risk for testicular cancer in ‘DES sons’, and for breast cancer after age 40, as well as a significant increase of vaginal adenocarcinomas in ‘DES daughters’. In a recent update after a 40 year follow up, the association between prenatal DES exposure and clear cell adenocarcinoma of the vagina and cervix was confirmed [50]. The teratogenic power of ionizing radiations and that of some drugs and, more generally, their ability to interact, even at low doses, with the fetal genome was already known in the 1950s, thanks to the studies (initially unfairly criticized) of Alice Stewart [51]. In more recent years, Lorenzo Tomatis experimentally demonstrated the direct link between the *in utero* exposure to DES of pregnant rats and the onset of (otherwise very rare) vaginal adenocarcinomas in unexposed offspring, including in the second and third generations [52]. On this basis, Tomatis also hypothesized that some childhood tumors could be the consequence of prenatal exposure to procarcinogenic chemicals [53]. In those years, epigenetics was only a theory and it was not even possible to hypothesize the molecular mechanisms originating the unexpected cases of transgenerational transmission of carcinogenic risk. Only after decades could scientists demonstrate that DES is a powerful endocrine disruptor that interferes with the expression of several uterine genes involved in tissue patterning, such as Wnt7a [54], Hoxa9, Hoxa10, and Hoxa11 [55], contributing to changes in the tissue architecture and morphology. The DES tragedy not only showed for the first time, the effects and, at least in part, the transgenerational carcinogenic mechanisms of a drug, but also documented that this could happen by altering the fetal programming of tissues and organs. We could also hypothesize that, within this model, the first hit in the process of carcinogenesis (tumor initiation) might occur already *in utero*. In the following years, scientists have shown that *in utero* exposure to DES can upturn the penetrance of a defective suppressor gene, while a second exposure (second hit) to analogous molecules could trigger the second step (tumor promotion) of the neoplastic process [56]. Moreover, the *in utero* exposure to endocrine disruptors predisposes both to neoplastic forms and to genitourinary malformations. This proves that the implicated molecular mechanisms are, at least in this early period of life, essentially epigenetic, and also that they are capable of perturbing cell differentiation and tissue development (fetal programming).

An important confirmation of the morphogenetic and carcinogenic potential of endocrine disruptors came from the studies that demonstrated that both TCDD [57] (the so-called Seveso dioxin recognized by the International Agency for Research on Cancer (IARC) as a potent carcinogen [58]) and PCBs [59] act in the fetus by altering the development of the mammary gland. The mammary gland is an organ characterized by a high degree of plasticity due to the various conformations and functions that it must fulfill across life and, particularly, during lactation [60]. It is not surprising that the tumors most directly related to endocrine disruptors, breast [61] and prostate cancer [62], have been among the most rapidly increasing in the world in the last few decades. 

Bisphenol-A (BPA) is another endocrine-disrupting chemical that can induce persistent epigenetic changes in the fetus, mainly in the developing uterus and breast. The molecular mechanisms by which epigenetic alterations would produce an increased risk of breast neoplasia after in utero exposure both to DES and BPA have been recently illustrated [63]. A model summarizing the main pathways potentially involved in the BPA action in prostate cancer was proposed by Di Donato et al., as follows: BPA could stimulate androgen receptor (AR)- and estrogen receptor (ER)-mediated gene transcription, contributing either to the enhancement or inhibition of cell proliferation. This could occur through epigenetic modifications such as those associated with the abnormal activity of histone-modifying enzymes (sirtuins, LSD/KDM lysine-demethylases), recruited to chromatin by steroidal receptors [64]. Santangeli et al. also proposed that the effects of BPA on female reproductive function could involve an epigenetic mechanism. The inhibitory action of BPA on the ovary could be due to its capacity to down-regulate the expression of luteinizing hormone/choriogonadotropin receptor (LHCGR), both decreasing and increasing H3K4me3 and H3K27me3, and interfering with DNMTs [65]. In conclusion, the most powerful procarcinogenic endocrine disruptors (molecules increasingly widespread throughout the whole ecosphere and in all food chains), seem to interfere epigenetically with the embryo-fetal programming of tissues and organs [66], thereby altering the regulation of genes involved in cell cycle, cell proliferation, apoptosis, and other key signaling pathways [67], rather than inducing stochastic mutations.

### 1.4. Environmental Exposures and Carcinogenesis

It has been demonstrated that small amounts of toxic pollutants, ionizing radiation, and possibly electromagnetic fields (EMFs) and their synergic action [68] may interfere with the epigenetic programming of fetal cells and tissues, which is one of the main causes of the continuous increase of chronic–degenerative, inflammatory, and neoplastic pathologies (DOHaD—Developmental origins of Health and Diseases) [69]. As for ionizing radiation, a clear epidemiological confirmation came from the KiKK study, which reported a 1.6-fold increase in solid cancers and a 2.2-fold increase in leukemia among children living within 5 km of all German nuclear power stations, because of the radionuclides released by reactors and incorporated by the gametes and/or directly by the fetuses (as clearly suggested by the increase of ‘embryonic’ tumors). The most reliable hypothesis is that the small quantities of radionuclides constantly emitted from the reactors had accumulated in the food chains [70]. For this reason, the increase was not recorded in the first generation exposed, but in subsequent generations. According to some authors, something similar is happening all over Europe after the Chernobyl accident. Also, in this case, the most probable mechanism could be represented by the epigenetic marks found in the gametes and transmitted from one generation to the next [71]. In addition to the increased incidence of thyroid cancers in the children not directly exposed, as a result of the exposure of the parental gametes [72], further evidence came from the observation that high rates of microsatellite mutations were observed in the children of the Chernobyl liquidators [73]. This also demonstrates that an epigenetic dysregulation underlies radiation-induced transgenerational genome instability [74]. Recently, Wakeford reviewed more than 30 studies worldwide, and confirmed the risks of exposure to radiation *in utero* (that is, the radiosensitivity of embryos and fetuses) [75].

### 1.5. Epidemiological Observations and Theoretical Models: The Epigenetic Paradigm Fits Better

What we have described so far needs a radical change, both in the dominant model of carcinogenesis and in the current pathogenic models concerning the increasing chronic diseases in the world. It is perhaps too early to propose this ‘dysontogenic model’ as the basic one to replace the still dominant somatic mutation theory in the field of human carcinogenesis. Still, it is worthwhile to propose some considerations regarding this complex issue.

Cancer prevalence has increased significantly worldwide over the past decades, becoming the most common chronic and life-threatening pathology (it is universally known that about one person out of two among the current inhabitants of our planet will develop sooner or later a malignant tumor). Crucially, it is also spreading more rapidly among young people and especially among children and adolescents [76], rather than in the elderly, as in the last century. These epidemiological trends would already be sufficient to undermine the dominant paradigm, based on the theory of the accumulation of stochastic mutations selected as advantageous for the cellular clone. Furthermore, the increase and anticipation in the age of diagnosis is underway for many other chronic diseases—endocrine and metabolic (the increase of obesity and type-2 diabetes is likely to be the biggest pandemic in human history) [77], immuno-inflammatory (allergies, autoimmune diseases), neurodevelopmental disorders (pervasive developmental disorders, schizophrenia, depression), and even neurodegenerative diseases (especially, Alzheimer’s disease), for which an early epigenetic origin has been also assumed [78]. This observation is in full consonance with the theory of the fetal/epigenetic origins of adult diseases (DOHaD), that in recent years, has been transformed from a pathogenic hypothesis to a general theory allowing a unitary and convincing interpretation of the ongoing epidemiological transition. [2]. DOHaD has been able to link the increase of metabolic and cardiovascular pathologies to different conditions of fetal distress (more and more frequently, as evidenced by the continuous increase in placental alterations, stillbirths, and preterm births) [79].

We propose that adopting this new the paradigm would help us in understanding the dramatic modifications in the incidence and prevalence of different forms of cancer, and above all, the mechanisms by which an increasingly widespread early exposure to pollution may determine them.

A fundamental corollary of this is the inadequacy of the current approaches in the field of environmental carcinogenesis. Indeed, if cancer and all other chronic inflammatory and degenerative diseases are increasing around the world among young people, this is a result of the anticipation of the exposure to the environmental factors that disturb the epigenetic programming in millions of embryos and fetuses; traditional epidemiological and toxicological studies are not an adequate way for studying and evaluating this phenomenon. The epidemiological studies present an important limitation, because the most serious and widespread effects of this increasingly precocious exposure are destined to manifest after decades and often only in the following generations [80]. For the toxicological studies, it is increasingly evident that the correlation between the exposure to toxic agents and its effects should be reconsidered as the small doses are often—epigenetically—more dangerous than the large ones (the critical windows of exposure being by far the most significant factor) [81]. Moreover, the real effects on the population will only be evident after decades (or even in future generations).

## 2. Conclusions

The epigenetic paradigm for carcinogenesis represents a valuable model, which recognizes cancer as a sometimes early (dysembryonic tumors), sometimes late consequence of a disrupted embryo-fetal development. It would allow us to recognize at the origin of cancer, a sequence of reactive-adaptive molecular and systemic mechanisms (potentially defensive) to multiple stressors perceived by the organism as dangerous. As in other pathologies, the embryo-fetal exposure to environmental, stressful, and proinflammatory triggers (first hit), seems to act as a ‘disease primer’, making fetal cells and tissues more susceptible to the subsequent environmental exposures (second hit), triggering the carcinogenic pathways. It is not uncommon that a better understanding of a pathology (and the related pathogenic processes) allows for the comprehension of physiological processes. In this case, proposing a more adequate carcinogenetic model would also contribute to unveiling the need for a paradigm shift in biomedicine. Eventually, we could emphasize that such an epigenetic and reactive model could allow us to adopt, also in the carcinogenic field, primary prevention strategies. These should be essentially based on the reduction of maternal-fetal and child exposure to several procarcinogenic agents and factors dispersed in the environment (as recently suggested by the World Health Organization) and in the food-chains, that seem to be at the origin of the continuous increase of neoplastic, inflammatory, and degenerative pathologies. The adoption of an epigenetic paradigm would also contribute to find out innovative ‘informational’ therapeutic strategies [82], like those successfully adopted in some highly malignant neoplastic diseases in early childhood [83]. 

## References

[1] Heindel J.J., Vandenberg L.N. (2015). Developmental Origins of Health and Disease: A Paradigm for Understanding Disease Etiology and Prevention. Curr. Opin. Pediatr..

[2] Burgio E. (2015). Environment and Fetal Programming: The origins of some current “pandemics”. J. Pediatr. Neonat. Individ. Med..

[3] Steliarova-Foucher E., Colombet M., Ries L.A.G., Moreno F., Dolya A., Bray F., Hesseling P., Shin H.Y., Stiller C.A. (2017). IICC-3 contributors. International incidence of childhood cancer, 2001-10: A population-based registry study. Lancet Oncol..

[4] Michor F., Iwasa Y., Nowak M.A. (2004). Dynamics of cancer progression. Nat. Rev. Cancer.

[5] Vineis P., Berwick M. (2006). The population dynamics of cancer: A Darwinian perspective. Int. J. Epidemiol..

[6] Vineis P., Melnick R. (2008). A Darwinian perspective: Right premises, questionable conclusion. A commentary on Niall Shanks and Rebecca Pyles’ “Evolution and medicine: The long reach of “Dr. Darwin””. Philos. Ethics Hum. Med..

[7] Wilkins A.S. (2008). Waddington’s Unfinished Critique of Neo-Darwinian Genetics: Then and Now. Biol. Theory.

[8] Shapiro J.A. (2009). Revisiting the central dogma in the 21st century. Ann. N. Y. Acad. Sci..

[9] Arthur W. (2002). The emerging conceptual framework of evolutionary developmental biology. Nature.

[10] Beldade P., Koops K., Brakefield P.M. (2002). Developmental constraints versus flexibility in morphological evolution. Nature.

[11] Prehn R.T. (1994). Cancers beget mutations versus mutations beget cancer. Cancer Res..

[12] Timp W., Feinberg A.P. (2013). Cancer as a dysregulated epigenome allowing cellular growth advantage at the expense of the host. Nat. Rev. Cancer.

[13] Korniluk A., Koper O., Kemona H., Dymicka-Piekarska V. (2017). From inflammation to cancer. IRISH J. Med. Sci..

[14] Mueller M.M., Fusenig N.E. (2004). Friends or foes—Bipolar effects of the tumour stroma in cancer. Nat. Rev. Cancer.

[15] Mbeunkui F., Johann D.J. (2009). Cancer and the tumor microenvironment: A review of an essential relationship. Cancer Chemother. Pharmacol..

[16] Streubel B., Chott A., Huber D., Exner M., Jäger U., Wagner O., Schwarzinger I. (2004). Lymphoma-specific genetic aberrations in microvascular endothelial cells in B-cell lymphomas. N. Engl. J. Med..

[17] Chen J.J., Lin Y.C., Yao P.L., Yuan A., Chen H.Y., Shun C.T., Tsai M.F., Chen C.H., Yang P.C. (2005). Tumor-associated macrophages: The double-edged sword in cancer progression. J. Clin. Oncol..

[18] Soto A.M., Sonnenschein C. (2005). Emergentism as a default: Cancer as a problem of tissue organization. J. Biosci..

[19] Soto A.M., Maffini M.V., Sonnenschein C. (2008). Neoplasia as development gone awry: The role of endocrine disruptors. Int. J. Androl..

[20] Heng H.H. (2008). The gene-centric concept: A new liability?. BioEssays.

[21] Feinberg A.P., Ohlsson R., Henikoff S. (2006). The epigenetic progenitor origin of human cancer. Nat. Rev. Genet..

[22] Goodson W.H., Lowe L., Carpenter D.O., Gilbertson M., Manaf Ali A., Lopez de Cerain A.S., Lasfar A., Carnero A., Azqueta A., Amedei A. (2015). Assessing the carcinogenic potential of low-dose exposures to chemical mixtures in the environment: The challenge ahead. Carcinogenesis.

[23] Duesberg P., Rasnick D. (2000). Aneuploidy, the somatic mutation that makes cancer a species of its own. Cell Motil. Cytoskel..

[24] Howe J.R., Klimstra D.S., Cordon-Cardo C., Paty P.B., Park P.Y., Brennan M.F. (1997). K-*ras* mutation in adenomas and carcinomas of the ampulla of Vater. Clin. Cancer Res..

[25] Jones P.A., Laird P.W. (1999). Cancer epigenetics comes of age. Nat. Genet..

[26] Jelinic P., Shaw P. (2007). Loss of imprinting and cancer. J. Pathol..

[27] Hauptmann S., Schmitt W.D. (2006). Transposable elements—Is there a link between evolution and cancer?. Med. Hypotheses.

[28] Cheah M.S., Wallace C.D., Hoffman R.M. (1984). Hypomethylation of DNA in human cancer cells: A site-specific change in the *c-myc* oncogene. J. Natl. Cancer Inst..

[29] Kulis M., Esteller M. (2010). DNA methylation and cancer. Adv. Genet..

[30] Luczak M.W., Jagodziński P.P. (2006). The role of DNA methylation in cancer development. Folia Histochem. Cytobiol..

[31] Herman J.G., Baylin S.B. (2003). Gene silencing in cancer in association with promoter hypermethylation. N. Engl. J. Med..

[32] Geutjes E.J., Bajpe P.K., Bernards R. (2012). Targeting the epigenome for treatment of cancer. Oncogene.

[33] Zhang B., Pan X., Cobb G.P., Anderson T.A. (2007). MicroRNAs as oncogenes and tumor suppressors. Dev. Biol..

[34] Vogelstein B., Kinzler K.W. (2004). Cancer genes and the pathways they control. Nat. Med..

[35] Couzin-Frankel J. (2015). Biomedicine. The bad luck of cancer. Science.

[36] Hanahan D., Weinberg R.A. (2000). De kenmerken van kanker. Cell.

[37] Hanahan D., Weinberg R.A. (2011). Hallmarks of Cancer: The Next Generation. Cell.

[38] Pavlova N.N., Thompson C.B. (2016). The emerging hallmarks of cancer metabolism. Cell Metab..

[39] Lazebnik Y. (2010). What are the hallmarks of cancer?. Nat. Rev. Cancer.

[40] Jolly M.K., Ware K.E., Gilja S., Somarelli J.A., Levine H. (2017). EMT and MET: Necessary or permissive for metastasis?. Mol. Oncol..

[41] Levin H.L., Moran J.V. (2011). Dynamic interactions between transposable elements and their hosts. Nat. Rev. Genet..

[42] Slamon D.J., Cline M.J. (1984). Expression of cellular oncogenes during embryonic and fetal development of the mouse. Proc. Natl. Acad. Sci. USA.

[43] Artandi S.E., DePinho R.A. (2010). Telomeres and telomerase in cancer. Carcinogenesis.

[44] Rangasamy D., Lenka N., Ohms S., Dahlstrom J.E., Blackburn A.C., Board P.G. (2015). Activation of LINE-1 Retrotransposon Increases the Risk of Epithelial-Mesenchymal Transition and Metastasis in Epithelial Cancer. Curr. Mol. Med..

[45] Hong W.S., Shpak M., Townsend J.P. (2015). Inferring the Origin of Metastases from Cancer Phylogenies. Cancer Res..

[46] Zhao Z.M., Zhao B., Bai Y., Iamarino A., Gaffney S.G., Schlessinger J., Lifton R.P., Rimm D.L., Townsend J.P. (2016). Early and multiple origins of metastatic lineages within primary tumors. Proc. Natl. Acad. Sci. USA.

[47] Gomez K., Miura S., Huuki L.A., Spell B.S., Townsend J.P., Kumar S. (2018). Somatic evolutionary timings of driver mutations. BMC Cancer.

[48] Burgio E., Migliore L. (2015). Towards a systemic paradigm in carcinogenesis: Linking epigenetics and genetics. Mol. Biol. Rep..

[49] Pierce G.B., Varney E.I. (1961). An in vitro and in vivo study of differentiation in teratocarcinomas. Cancer.

[50] Huo D., Anderson D., Palmer J.R., Herbst A.L. (2017). Incidence rates and risks of diethylstilbestrol-related clear-cell adenocarcinoma of the vagina and cervix: Update after 40-year follow-up. Gynecol. Oncol..

[51] Stewart A.M., Webb J.W., Giles B.D., Hewitt D. (1956). Preliminary Communication: Malignant Disease in Childhood and Diagnostic Irradiation In Utero. Lancet.

[52] Yamasaki H., Loktionov A., Tomatis L. (1992). Perinatal and multigenerational effect of carcinogens: Possible contribution to determination of cancer susceptibility. Environ. Health Perspect..

[53] Tomatis L. (1979). Prenatal exposure to chemical carcinogens and its effect on subsequent generations. Natl. Cancer Inst. Monogr..

[54] Miller C., Degenhardt K., Sassoon D.A. (1998). Fetal exposure to DES results in de-regulation of Wnt7a during uterine morphogenesis. Nat. Genet..

[55] Block K., Kardana A., Igarashi P., Taylor H.S. (2000). *In utero* diethylstilbestrol (DES) exposure alters Hox gene expression in the developing mullerian system. FASEB J..

[56] Cook J.D., Davis B.J., Cai S.L., Barrett J.C., Conti C.J., Walker C.L. (2005). Interaction between genetic susceptibility and early-life environmental exposure determines tumor-suppressor-gene penetrance. Proc. Natl. Acad. Sci. USA.

[57] Vorderstrasse B.A., Fenton S.E., Bohn A.A., Cundiff J.A., Lawrence B.P. (2004). A novel effect of dioxin: Exposure during pregnancy severely impairs mammary gland differentiation. Toxicol. Sci..

[58] Steenland K., Bertazzi P., Baccarelli A., Kogevinas M. (2004). Dioxin Revisited: Developments Since the 1997 IARC Classification of Dioxin as a Human Carcinogen. Environ. Health Perspect..

[59] Moral R., Wang R., Russo I.H., Lamartiniere C.A., Pereira J., Russo J. (2008). Effect of prenatal exposure to the endocrine disruptor bisphenol a on mammary gland morphology and gene expression signature. J. Endocrinol..

[60] Gray J.M., Rasanayagam S., Engel C., Rizzo J. (2017). State of the evidence 2017: An update on the connection between breast cancer and the environment. Environ. Health.

[61] Giulivo M., Lopez de Alda M., Capri E., Barceló D. (2016). Human exposure to endocrine disrupting compounds: Their role in reproductive systems, metabolic syndrome and breast cancer. A review. Environ. Res..

[62] Prins G.S., Tang W.Y., Belmonte J., Ho S.M. (2008). Perinatal exposure to oestradiol and bisphenol A alters the prostate epigenome and increases susceptibility to carcinogenesis. Basic Clin. Pharmacol. Toxicol..

[63] Doherty L.F., Bromer J.G., Zhou Y., Aldad T.S., Taylor H.S. (2010). In utero exposure to diethylstilbestrol (DES) or bisphenol-A (BPA) increases EZH2 expression in the mammary gland: An epigenetic mechanism linking endocrine disruptors to breast cancer. Horm Cancer.

[64] Di Donato M., Cernera G., Giovannelli P., Galasso G., Bilancio A., Migliaccio A., Castoria G. (2017). Recent advances on bisphenol-A and endocrine disruptor effects on human prostate cancer. Mol. Cell. Endocrinol..

[65] Santangeli S., Maradonna F., Olivotto I., Piccinetti C.C., Gioacchini G., Carnevali O. (2017). Effects of BPA on female reproductive function: The involvement of epigenetic mechanism. Gen. Comp. Endocrinol..

[66] Del Pup L., Mantovani A., Cavaliere C., Facchini G., Luce A., Sperlongano P., Caraglia M., Berretta M. (2016). Carcinogenetic mechanisms of endocrine disruptors in female cancers. Oncol Rep..

[67] Bernal A.J., Jirtle R.L. (2010). Epigenomic disruption: The effects of early developmental exposures: Epigenomic reactions to early exposures. Birth Defects Res. Part A Clin. Mol. Teratol..

[68] Manti L., D’Arco A. (2010). Cooperative biological effects between ionizing radiation and other physical and chemical agents. Mutat. Res..

[69] Barouki R., Melén E., Herceg Z., Beckers J., Chen J., Karagas M., Puga A., Xia Y., Chadwick L., Yan W. (2018). Epigenetics as a mechanism linking developmental exposures to long-term toxicity. Environ. Int..

[70] Fairlie I. (2009). Commentary: Childhood cancer near nuclear power stations. Environ. Health.

[71] Busby C.C. (2009). Very low dose fetal exposure to Chernobyl contamination resulted in increases in infant leukemia in Europe and raises questions about current radiation risk models. Int. J. Environ. Res. Public Health.

[72] Williams D. (2002). Cancer after nuclear fallout: Lessons from the Chernobyl accident. Nat. Rev. Cancer.

[73] Weinberg H.S., Kovol A.B., Kirzher V.M. (2001). Very high mutation rates in offspring of Chernobyl accident liquidators. Proc. R. Soc. Lond..

[74] Koturbash I., Baker M., Loree J., Kutanzi K., Hudson D., Pogribny I., Sedelnikova O., Bonner W., Kovalchuk O. (2006). Epigenetic dysregulation underlies radiation-induced transgenerational genome instability in vivo. Int. J. Radiat. Oncol. Biol. Phys..

[75] Wakeford R. (2008). Childhood leukemia Following Medical Diagnostic Exposure to Ionising Radiation in Utero or after Birth. Radiat. Prot. Dosimetry.

[76] Pritchard-Jones K., Kaatsch P., Steliarova-Foucher E., Stiller C., Coebergh J.W. (2006). Cancer in children and adolescents in Europe. Eur. J. Cancer.

[77] Zimmet P.Z. (2017). Diabetes and its drivers: The largest epidemic in human history?. Clin. Diabetes Endocrinol..

[78] Lahiri D.K., Maloney B., Basha M.R., Ge Y.W., Zawia N.H. (2007). How and when environmental agents and dietary factors affect the course of Alzheimer’s disease: The “LEARn” model (latent early-life associated regulation) may explain the triggering of AD. Curr. Alzheimer Res..

[79] Lawn J.E., Blencowe H., Waiswa P., Amouzou A., Mathers C., Hogan D., Flenady V., Frøen J.F., Qureshi Z.U., Calderwood C. (2016). Lancet Ending Preventable Stillbirths Series study group; Lancet Stillbirth Epidemiology investigator group. Stillbirths: Rates, risk factors, and acceleration towards 2030. Lancet.

[80] Braun J.M., Gray K. (2017). Challenges to studying the health effects of early life environmental chemical exposures on children’s health. PLoS Biol..

[81] Selevan S.G., Kimmel C.A., Mendola P. (2000). Identifying critical windows of exposure for children’s health. Environ. Health Perspect..

[82] Lang J.Y., Shi Y., Chin Y.E. (2013). Reprogramming cancer cells: Back to the future. Oncogene.

[83] Degos L., Wang Z.Y. (2001). All trans-retinoic acid in acute promyelocytic leukemia. Oncogene.

